# Testosterone propionate activated the Nrf2-ARE pathway in ageing rats and ameliorated the age-related changes in liver

**DOI:** 10.1038/s41598-019-55148-0

**Published:** 2019-12-09

**Authors:** Guoliang Zhang, Rui Cui, Yunxiao Kang, Chunxiao Qi, Xiaoming Ji, Tianyun Zhang, Qiqing Guo, Huixian Cui, Geming Shi

**Affiliations:** 1 Department of Neurobiology, Hebei Medical University, Shijiazhuang, 050017 P.R. China; 2 Department of Human Anatomy, Hebei Medical University, Shijiazhuang, 050017 P.R. China; 3 Neuroscience Research Center, Hebei Medical University, Shijiazhuang, 050017 P.R. China; 4 Hebei Key Laboratory of Forensic Medicine, Department of Forensic Medicine, Hebei Medical University, Shijiazhuang, 050017 P.R. China

**Keywords:** Liver fibrosis, Hepatocytes

## Abstract

The present study aimed to evaluate the protective efficacy of testosterone propionate (TP) on age-related liver changes via activation of the nuclear factor erythroid 2-related factor 2-antioxidant response element (Nrf2-ARE) pathway in aged rats. Aged rats received subcutaneous injections of TP (2 mg/kg/d, 84 days). Oxidative stress parameters and the expression levels of signal transducer and activator of transcription 5b (STAT5b), Kelch-like ECH associating protein-1 (Keap1), Nrf2, haem oxygenase-1 (HO-1) and NAD(P)H: quinone oxidoreductase-1 (NQO1) in liver tissues were examined to check whether the Nrf2-ARE pathway was involved in the age-related changes in liver. Our results showed that TP supplementation alleviated liver morphology, liver function and liver fibrosis; improved oxidative stress parameters; and increased the expression of STAT5b, Nrf2, HO-1 and NQO-1 and decreased the expression of Keap1 in the liver tissues of aged rats. These results suggested that TP increased the expression of STAT5b, and then activated the Nrf2-ARE pathway and promoted antioxidant mechanisms in aged rats. These findings may provide new therapeutic uses for TP in patients with age-related liver changes.

## Introduction

Ageing is characterized by a process of physiological degeneration and is accompanied by pathophysiological age-related changes, including impaired functioning and increased vulnerability to death^1–3^. Age-related changes in the liver are commonly associated with structural and physiological changes^4,5^. The mechanisms of hepatic injury caused by ageing are complicated and involve apoptosis, inflammation, hepatic clearance function and toxicity^6–8^. Recent studies have shown that oxidative stress is one of the mechanisms implicated in the progression of age-related changes in the liver^9,10^. In this way, antioxidants could be important therapeutic strategies for controlling such changes in the liver.

Nuclear factor erythroid-related factor 2 (Nrf2), an important antioxidant transcription factor, regulates the expression of many antioxidant and phase II detoxifying enzyme genes, such as haem oxygenase-1 (HO-1) and NADP(H): quinine oxidoreductase-1 (NQO1), through the antioxidant response element (ARE). Nrf2 is repressed in the cytoplasm by the Kelch-like ECH associating protein-1 (Keap1) under normal conditions. However, Nrf2 dissociates from Keap1 and translocates to the nucleus to bind to ARE upon oxidative stress^11^. Activation of the Nrf2-ARE pathway has a protective effect against various diseases via antioxidative mechanisms^12–15^.

Testosterone is an important hormone that participates in a variety of physiological functions, including embryonic development^16^, stimulation of genital growth and secondary sexual characteristics^17^, maintenance of spermatogenesis^18^, improved sexual function^19^ and promotion of erythropoiesis^20^. It has been reported that concentrations of testosterone decrease during the ageing process^21,22^. The level of growth hormone (GH) show the age-related reduction, and testosterone increases GH secretion in older men^23^. Signal transducer and activator of transcription 5b (STAT5b), responds to a variety of extracellular cytokine and growth factor signals, including GH^24^. Regulation of STAT5b through GH secretion is affected by physiological stimulators or inhibitors^24,25^. Furthermore, testosterone has been reported to play a protective role by activating the Nrf2-ARE pathway in dopaminergic neurons in the substantia nigra of aged rats^26^. However, whether testosterone has similar effects on liver tissue has not been explored. In the present study, changes in the liver morphology and function of aged rats after testosterone propionate (TP) treatment were observed, and we determined whether TP administration activated the Nrf2-ARE pathway to exert protective effects against age-related changes in the liver.

## Results

### The effect of TP on liver morphology in aged rats

**Figure 1:**
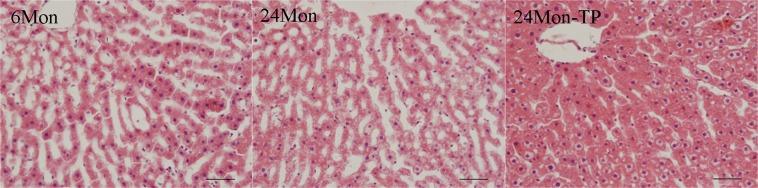
H&E staining of liver tissues. The 24 Mon group rats showed morphological changes, including fuzzy liver cell morphological structure, liver cell hypertrophy and connective tissue hyperplasia. TP supplementation improved ageing-induced hepatic injury. Bar = 100 μm.

The 6 Mon group rats showed regular liver morphology with no evidence of histopathological changes. The 24 Mon group rats showed fuzzy liver cell morphological structure, liver cell hypertrophy and connective tissue hyperplasia. In contrast, TP supplementation improved ageing-induced hepatic injury (Fig. 1).

### The effect of TP on liver function in aged rats

**Figure 2:**
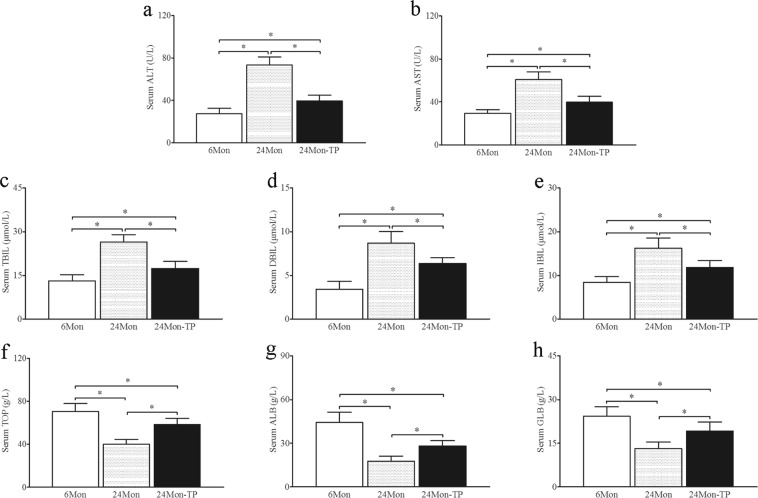
The effect of TP on liver function in aged rats. Bar graphs show the serum levels of alanine transaminase (ALT) (**a**), aspartate transaminase (AST) (**b**), total bilirubin (TBIL) (**c**), direct bilirubin (DBIL) (**d**), indirect bilirubin (IBIL) (**e**), total protein (TOP) (**f**), albumin (ALB) (**g**) and globulin (GLB) (**h**). The asterisks show significant differences (*P < 0.01).

The levels of alanine transaminase (ALT, Fig. 2a), aspartate transaminase (AST, Fig. 2b), total bilirubin (TBIL, Fig. 2c), direct bilirubin (DBIL, Fig. 2d) and indirect bilirubin (IBIL, Fig. 2e) in 24 Mon rats were significantly increased compared to those in 6 Mon rats (*P* < 0.01). The levels of total protein (TOP, Fig. 2f), albumin (ALB, Fig. 2g) and globulin (GLB, Fig. 2h) in 24 Mon rats were significantly decreased compared to those in 6 Mon rats (*P* < 0.01). The levels of ALT, AST, TBIL, DBIL and IBIL of 24 Mon-TP rats were decreased, and the levels of TOP, ALB and GLB of 24 Mon-TP rats were increased after TP treatment compared with those of untreated 24 Mon rats (*P* < 0.01). However, the serum levels of ALT, AST, TBIL, DBIL, IBIL, TOP, ALB and GLB of 24 Mon-TP rats were not restored to the corresponding levels of 6 Mon rats (*P* < 0.01) (Fig. 2).

### The effect of TP on liver fibrosis in aged rats

**Figure 3:**
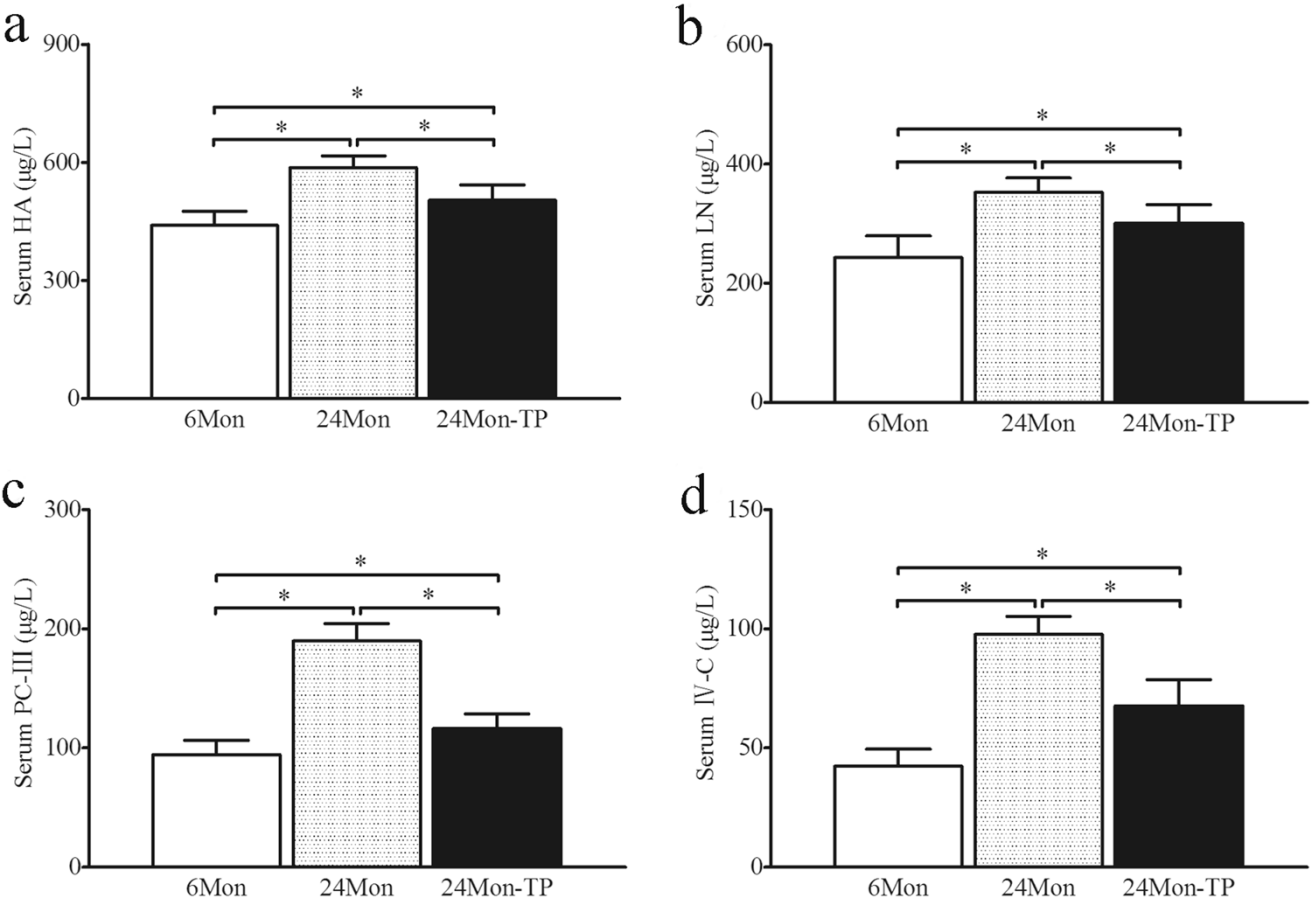
The effect of TP on liver fibrosis in aged rats. Bar graphs show the serum levels of hyaluronidase (HA) (**a**), laminin (LN) (**b**), type III precollagen (PC-III) (**c**) and type IV collagen (IV-C) (**d**). The asterisks show significant differences (*P < 0.01).

The levels of hyaluronidase (HA, Fig. 3a), laminin (LN, Fig. 3b), type III precollagen (PC-III, Fig. 3c) and type IV collagen (IV-C, Fig. 3d) in 24 Mon rats were significantly increased compared to those in 6 Mon rats (*P* < 0.01). The levels of HA, LN, PC-III and IV-C in 24 Mon-TP rats were significantly decreased after TP treatment compared with those in 24 Mon rats (*P* < 0.01) and were not restored to the levels in 6 Mon rats (*P* < 0.01) (Fig. 3).

### The effect of TP on serum growth hormone (GH) in aged rats

The level of serum GH in 24 Mon rats (0.4 ± 0.1 μg/L) was significantly decreased compared to that in 6 Mon rats (1.8 ± 0.2 μg/L*, P* < 0.01). The level of serum GH in 24 Mon-TP rats (1.4 ± 0.2 μg/L) was significantly increased after TP treatment compared with that in 24 Mon rats (*P* < 0.01) and was not restored to the level in 6 Mon rats (*P* < 0.01).

### The effect of TP on oxidative stress parameters in aged rats

**Figure 4:**
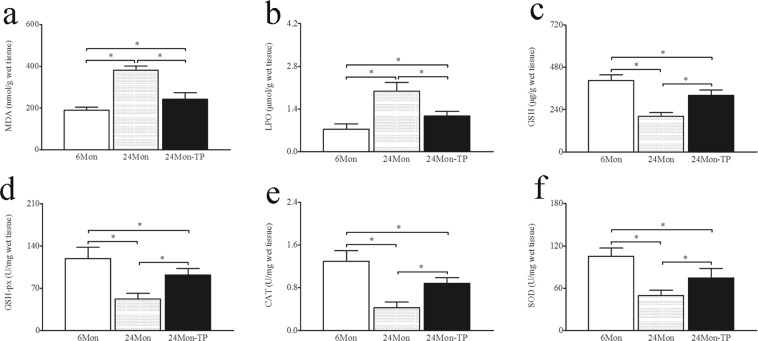
The effect of TP on oxidative stress parameters in the liver tissue of aged rats. Bar graphs show the levels of malondialdehyde (MDA) (**a**), lipid peroxidation (LPO) (**b**), reduced glutathione (GSH) (**c**), glutathione peroxidase (GSH-px) (**d**), catalase (CAT) (**e**) and superoxide dismutase (SOD) (**f**) in the liver tissue. The asterisks show significant differences (*P < 0.01).

The levels of malondialdehyde (MDA, Fig. 4a) and lipid peroxidation (LPO, Fig. 4b) in 24 Mon rats were significantly increased compared to those in 6 Mon rats (*P* < 0.01), and the levels of reduced glutathione (GSH, Fig. 4c), glutathione peroxidase (GSH-px, Fig. 4d), catalase (CAT, Fig. 4e) and superoxide dismutase (SOD, Fig. 4f) in 24 Mon rats were significantly decreased compared to those in 6 Mon rats (*P* < 0.01). Notably, the levels of MDA and LPO in 24 Mon-TP rats were decreased, and the levels of GSH, GSH-px, CAT and SOD in 24 Mon-TP rats were increased after TP treatment compared to those in 24 Mon rats (*P* < 0.01). The serum levels of MDA, LPO, GSH, GSH-px, CAT and SOD in 24 Mon-TP rats were not restored to the corresponding levels in 6 Mon rats (*P* < 0.01) (Fig. 4).

### The effect of TP on the STAT5b, Keap1, Nrf2-ARE pathway in aged rats

#### STAT5b, Keap1, Nrf2, HO-1 and NQO-1 mRNA expression

**Figure 5:**
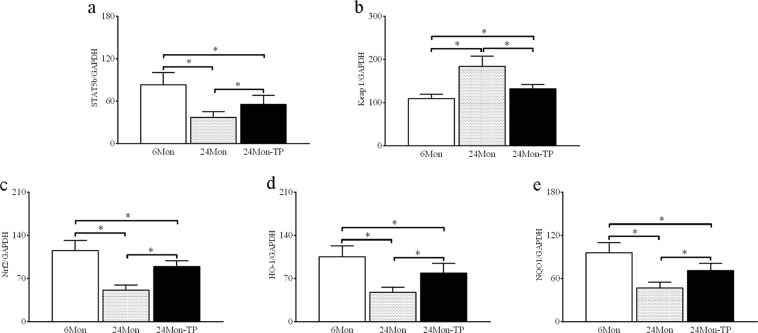
The effect of TP on STAT5b, Keap1 and the Nrf2-ARE signalling pathway mRNA in the liver tissue of aged rats. Bar graphs show STAT5b mRNA (**a**), Keap1 mRNA (**b**), Nrf2 mRNA (**c**), HO-1 mRNA (**d**) and NQO1 mRNA (**e**). The asterisks show significant differences (*P < 0.01). STAT5b: signal transducer and activator of transcription 5b; Keap1: Kelch-like ECH associating protein-1; Nrf2: nuclear factor erythroid 2-related factor 2; HO-1: haem oxygenase-1; NQO1: NAD(P)H: quinone oxidoreductase-1.

The expression levels of STAT5b mRNA (Fig. 5a), Nrf2 mRNA (Fig. 5c), HO-1 mRNA (Fig. 5d) and NQO1 mRNA (Fig. 5e) in 24 Mon rats were significantly decreased compared to those in 6 Mon rats (*P* < 0.01), and the expression level of Keap1 mRNA (Fig. 5b) in 24 Mon rats was significantly increased compared to that in 6 Mon rats (*P* < 0.01). The expression levels of STAT5b mRNA, Nrf2 mRNA, HO-1 mRNA and NQO1 mRNA in 24 Mon-TP rats were increased compared with those in 24 Mon rats (*P* < 0.01), and the expression level of Keap1 mRNA in 24 Mon-TP rats was significantly decreased compared to that in 24 Mon rats (*P* < 0.01). The expression levels of STAT5b mRNA, Keap1 mRNA, Nrf2 mRNA, HO-1 mRNA and NQO1 mRNA in 24 Mon-TP rats were not restored to the levels in 6 Mon rats (*P* < 0.01) (Fig. 5).

#### STAT5b, Keap1, Nrf2, HO-1 and NQO-1 Protein expression

**Figure 6:**
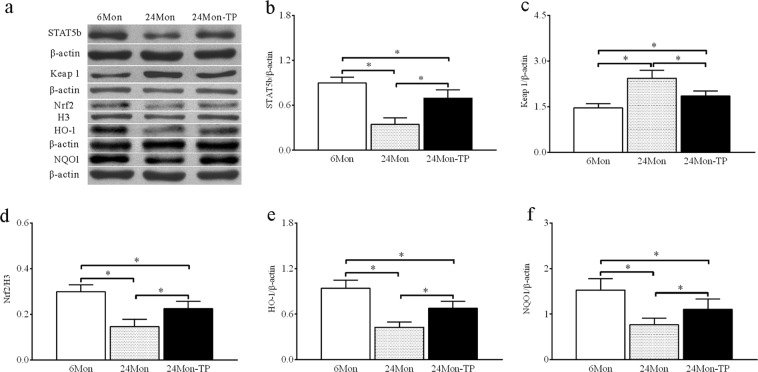
The effect of TP on STAT5b, Keap1 and the Nrf2-ARE signalling pathway proteins in the liver tissue of aged rats. Western blot analysis revealed STAT5b, Keap1, Nrf2, HO-1 and NQO1 proteins expression in liver tissue. (**a**) Bar graphs illustrate the protein expression of STAT5b (**a**), Keap1 (**b**), Nrf2 (**c**), HO-1 (**d**) and NQO1 (**e**). The asterisks show significant differences (*P < 0.01). STAT5b: signal transducer and activator of transcription 5b; Keap1: Kelch-like ECH associating protein-1; Nrf2: nuclear factor erythroid 2-related factor 2; HO-1: haem oxygenase-1; NQO1: NAD(P)H: quinone oxidoreductase-1.

Western blotting showed that STAT5b, Keap1, Nrf2, HO-1 and NQO1 proteins were located at ~90 kDa, ~70 kDa, ~68 kDa, ~32 kDa and ~30 kDa, respectively (Fig. 6a). The expression levels of STAT5b (Fig. 6b), Nrf2 (Fig. 6d), HO-1 (Fig. 6e) and NQO1 (Fig. 6f) proteins in 24 Mon rats were significantly decreased compared to those in 6 Mon rats (*P* < 0.01) and the expression level of Keap1 protein (Fig. 6c) in 24 Mon rats was significantly increased compared to that in 6 Mon rats (*P* < 0.01). The expression levels of STAT5b, Nrf2, HO-1 and NQO1 proteins in 24 Mon-TP rats increased compared with those in 24 Mon rats (*P* < 0.01), and the expression level of Keap1 protein in 24 Mon-TP rats was significantly decreased compared to that in 24 Mon rats (*P* < 0.01). The expression levels of STAT5b, Keap1, Nrf2, HO-1 and NQO1 proteins in 24 Mon-TP rats were not restored to the levels in 6 Mon rats (*P* < 0.01) (Fig. 6).

**Figure 7:**
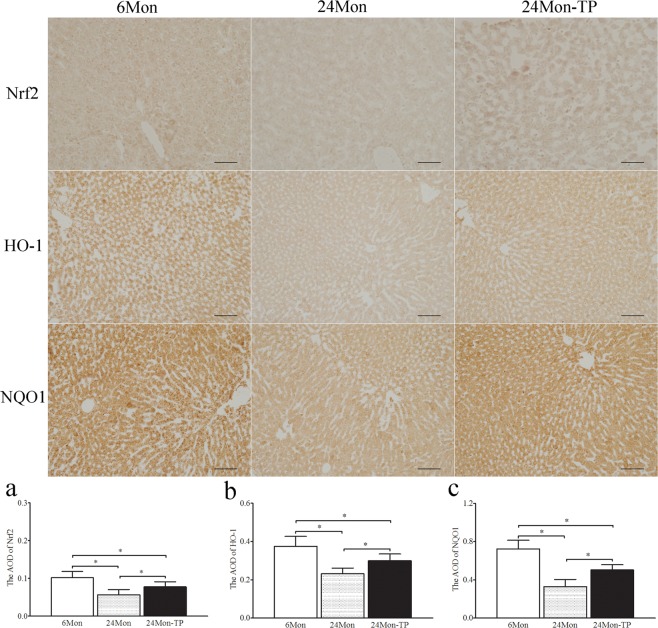
Immunohistochemistry revealed variations in Nrf2, HO-1 and NQO1 immunoreactive intensity in the liver tissue of aged rats. Bar = 200 μm. Bar graph shows the AOD of Nrf2 (**a**), HO-1 (**b**) and NQO1 (**c**). The asterisks show significant differences (*P < 0.01). AOD: average optical density; Nrf2: nuclear factor erythroid 2-related factor 2; HO-1: haem oxygenase-1; NQO1: NAD(P)H: quinone oxidoreductase-1.

Immunohistochemistry revealed variations in the immunoreactive intensity of Nrf2, HO-1 and NQO1. The AOD of Nrf2 (Fig. 7a), HO-1 (Fig. 7b) and NQO1 (Fig. 7c) in 24 Mon rats was significantly decreased compared to that in 6 Mon rats (*P* < 0.01). The AOD of Nrf2, HO-1 and NQO1 in 24 Mon-TP rats was increased compared with that in 24 Mon rats (*P* < 0.01) and was not restored to the levels in 6 Mon rats (*P* < 0.01) (Fig. 7).

## Discussion

In the present study, TP supplementation improved age-related liver morphological changes. The serum levels of ALT, AST, TBIL, DBIL, IBIL, TOP, ALB and GLB were used to explore liver function. Our data showed that TP treatment decreased ALT, AST, TBIL, DBIL and IBIL levels and increased TOP, ALB and GLB levels. The serum levels of HA, LN, PC-III and IV-C were used to explore liver fibrosis. These data showed that TP treatment resulted in a significant decrease in HA, LN, PC-III and IV-C levels. These results confirmed that TP supplementation alleviated changes in liver morphology, liver function and liver fibrosis in aged rats.

Accumulating evidence indicates that reactive oxygen species (ROS) regulate longevity in many organisms^27,28^. This free radical theory suggests that ageing is caused by an accumulation of molecular damage resulting from ROS, such as superoxide anions, hydroxyl radicals and hydrogen peroxide^29^. In the antioxidant defence system, Nrf2 is the most important transcription factor in regulating multiple antioxidants^30^. Activation of the Nrf2/HO-1 pathway might play a potential protective role against doxorubicin-induced hepatotoxicity^31^. Moreover, the preventive effect against CCl_4_-induced hepatotoxicity and fibrosis was partly dependent on modulation of the Nrf2-ARE signalling pathway^32^. The mechanism of the cytoprotective effect of rutaecarpine against tert-butyl hydroperoxide occurs via upregulating antioxidant enzymes in part via the Nrf2-ARE pathways^33^. Ethyl acetate treatment has been shown to significantly increase the levels of Nrf2 and inhibit CCl_4_-induced liver fibrosis in rats^34^. In this way, activation of the Nrf2-ARE signalling pathway has a protective effect on the liver. In our previous studies, intranasal testosterone propionate supplementation increased the levels of Nrf2, HO-1 and NQO1 and enhanced dopaminergic functional activity in the substantia nigra and ventral tegmental area^22^. However, the role of TP in peripheral liver tissues has not been reported.

STAT5b is downstream of GH signalling. With the pulsatile secretion of GH, this leads to the formation of STAT5b homodimers^24^. Whether STAT5b regulates the Keap1 and Nrf2-ARE pathway in the liver is unclear. Our data shows that the level of serum GH and the expression level of STAT5b were increased after TP treatment in aged rats. The levels of MDA, LPO, GSH, GSH-px, CAT and SOD in hepatic tissues represent the state of oxidative stress^35^. In our study, the levels of MDA and LPO were decreased, and the levels of GSH, GSH-px, CAT and SOD were increased in aged rats after TP treatment. Nrf2 is a key transcription factor that plays a crucial role in defending against oxidative stress through modulation of its downstream antioxidant and detoxifying enzymes^36^. The cytoplasmic genes HO-1 and NQO1 could be induced by Nrf2 nuclear translocation^37,38^. The expression levels of Nrf2 (nuclear), HO-1 and NQO1 (cytoplasm) were increased and the expression level of Keap1 (cytoplasm) was decreased after TP supplementation in aged rats. These results suggested that TP promotes GH secretion, increases the expression of STAT5b, induces the separation of Keap1 and Nrf2 in the cytoplasm and promotes Nrf2 transfer to the nucleus and bind to ARE, subsequently activates the Nrf2-ARE pathway and improves oxidative stress in aged hepatic tissues. Therefore, the protective effects of TP on aged liver tissues may partly depend on activation of the Nrf2-ARE signalling pathway followed by a decrease in ROS. The molecular mechanism of testosterone on age-related changes in the liver is not fully understood, and more detailed studies are needed to accurately identify the mechanism. Our results of TP on the activation of Nrf2 in the liver show some disparity with a previous study^25^. In male mice, Nrf2 was activated by ethinyl estradiol and was suppressed by dihydrotestosterone, whereas in female mice, Nrf2 was suppressed by testosterone and was activated by ethinyl estradiol^25^. Testosterone can be converted to dihydrotestosterone and estradiol by 5α-reductase and aromatase respectively in male rats^39^. Thus, we presume that the difference of our results with the previous study is the sum effects of dihydrotestosterone and estradiol. In the liver of aged male rats, testosterone might show efficacy of estradiol on Nrf2. This requires further investigation in future research. In addition, different species, different time of administration and dose and the status of oxidative stress in the liver might contribute to the difference.

In conclusion, the results of the present study suggested that TP increased the expression of STAT5b, and then activated Nrf2-ARE signalling and promoted antioxidant mechanisms in aged rats. TP treatment appeared to play the role of an Nrf2 activator and alleviate age-related changes in the liver. This finding may provide new therapeutic uses for TP in patients with age-related liver disease.

## Materials and Methods

### Animals and testosterone propionate supplement

Forty-eight male Wistar rats (Experimental Animal Center of Hebei Medical University) were divided into three groups consisting of a 6-month-old group (6 Mon), a TP-supplemented group (24 Mon-TP) and an aged vehicle control group (24 Mon). Each group included sixteen rats. The 24 Mon-TP group rats received a daily subcutaneous TP injection (2 mg/kg/d at 5:00 PM to 6:00 PM) beginning at the age of 21 months. Supplementation with TP was continued for 12 weeks (84 days). The 6 Mon group rats and 24 Mon group rats received a sesame oil injection (vehicle)^40^. All rats were housed in a room maintained at a constant temperature (22 ± 2 °C). All animal procedures were performed in accordance with the National Institutes of Health Guide for the Care and Use of Laboratory Animals and were approved by the Local Animal Use Committee of Hebei Medical University.

### Histopathologic evaluation

The eight rats in each group were anaesthetized by intraperitoneal injection of 4% chloral hydrate (300 mg/kg body weight) and transcardially perfused with saline. Then, the rats underwent perfusion with 4% paraformaldehyde in 0.1 M phosphate buffer (pH 7.4). Livers were removed, and tissue sections were post-fixed in 4% paraformaldehyde for 4 h (4 °C). The liver tissue sections of the rats were washed in two changes of fresh phosphate buffer, dehydrated in titrated ethanol and cleared in xylene before being embedded in paraffin wax. The blocks were sliced into sections at a 5 μm thickness on a sliding microtome (Leica-RM2145, Germany) and the sections were stained with H&E. The preparations were evaluated by light microscopy and photographed (Olympus, BX 61, Japan)^40^.

### Measurement of liver function in serum

Serum levels of ALT, AST, TBIL, DBIL, IBIL, TOP, ALB and GLB were measured with a fully automatic biochemical analyser (HITACHI-7180).

### Measurement of liver fibrosis indexes and GH in serum

Levels of serum HA, LN and GH were measured using an ELISA kit. Levels of serum PC-III and IV-C were analysed by radioimmunoassay (RIA) and determined from a standard curve. Kits for HA (FA02052B) and LN (FA01730B) were provided by the Shanghai Yantuo Biological Technology Co., LTD. Kits for PC-III (HY-10089) and IV-C (HY-10086) were purchased from Shanghai Huzhen Industrial Co., Ltd. Kit for GH (ARB12139) was purchased from Beijing Baiao Laibo Technology Co., Ltd. All steps were performed according to the manufacturers’ protocols.

### Measurement of oxidative stress parameters in liver

The liver tissue was homogenized separately with 10 times (w/v) ice-cold 0.1 M phosphate buffer (PB) at pH 7.4. The homogenates were used to assess oxidative stress parameters. MDA, LPO, GSH, GSH-px, CAT and SOD levels were measured spectrophotometrically using detection kits, following the manufacturer’s instructions (Nanjing Jiancheng Bioengineering Institute, China)^40^.

### Quantitative real-time polymerase chain reaction

Total RNA from the liver tissue was obtained using TRIzol reagent (Invitrogen, USA) following the instructions. The RNA concentration was determined by measuring the absorbance (A) of a diluted sample at the 260 nm wavelength in a UV spectrometer. A total of 2 µg of total RNA was subjected to reverse transcription using a random primer to obtain the first-strand cDNA template. Real-time fluorescence quantitative PCR was performed with 0.8 µl cDNA (diluted 1:10), 2 µl of specific primers, and 2*GoTaq® Green Master Mix (Promega, USA) in a final volume of 20 µl. PCR was performed as follows: an initial cycle at 95 °C for 10 min, followed by 40 cycles at 95 °C for 15 s, 58 °C for 20 s and 72 °C for 27 s. Then, PCR products were analysed by melting curve to confirm the specificity of amplification^40^. The expression levels of STAT5b, Keap1, Nrf2, HO-1 and NQO1 genes were analysed with GAPDH as an internal control. The sets of primers that we used were as follows: STAT5b (5′-AGCAGGCTTTTGGCATCAT-3′ and 5′-CCGTGTAGGCGAACTCAATTAG-3′), Keap1 (5′-GCAATGATTACAGCGGCGAGA-3′ and 5′-CAAAGGCGTTGTCCCAGAGG-3′), Nrf2 (5′-GACCTAAAGCACAGCCAACACAT-3′ and 5′-CTCAATCGGCTTGAATGTTTGTC-3′), HO-1 (5′-TGTCCCAGGATTTGTCCGAG-3′ and 5′-ACTGGGTTCTGCTTGTTTCGCT-3), NQO1 (5′-GGGGACATGAACGTCATTCTCT-3′ and 5′-AGTGGTGACTCCTCCCAGACAG-3′), and GAPDH (5′TGAACGGGAAGCTCACTG3′ and 5′GCTTCACCACCTTCTTGATG3′).

### Western blot analysis

The liver tissue was homogenized in ice-cold lysis buffer (10 mmol/L HEPES pH 7.9, 10 mmol/L KCl, 0.1 mmol/L EDTA, 1 mmol/L DTT, 0.1 mmol/L EGTA) for 15 min. After adding NP-40, the homogenate was centrifuged at 10,000 rpm at 4 °C for 3 min, and the supernatant was collected as cytoplasmic protein for STAT5b, Keap1, HO-1 and NQO1. The pellets were homogenized in ice-cold lysis buffer (20 mmol/L HEPES, pH 7.9, 400 mmol/L NaCl, 1 mmol/L EDTA, 0.1 mmol/L EGTA) for 15 min. Then, the pellets were centrifuged at 12,000 rpm at 4 °C for 10 min, and the supernatant was collected. Phenylmethanesulfonyl fluoride was added to the supernatant at a final concentration of 1 mmol/L as the nuclear protein for Nrf2^40^.

The protein concentration of the supernatant was determined using a BCA Protein Assay reagent kit (Novagen, Madison, WI, USA). Liver tissue (50 µg) protein samples were separated by SDS/PAGE and transferred to PVDF membranes. Membranes were blocked with 5% skimmed milk for 1 h at room temperature and then probed with monoclonal mouse anti-Nrf2 antibody (1:100, Abcam, ab89443), monoclonal rabbit anti-STAT5b antibody (1:5000, Abcam, ab178941), polyclonal rabbit anti-Keap1 antibody (1:500, Abcam, ab139729), polyclonal rabbit anti-HO-1 antibody (1:200, Abcam, ab13243) and polyclonal rabbit anti-NQO1 antibody (1:200, Abcam, ab34173) overnight at 4 °C. After washing three times with phosphate-buffered saline with 1% Tween 20, the membranes were incubated with IRDye® 800-conjugated goat anti-rabbit second antibody (1:3000, Rockland, Gilbertsville) for 1 h at room temperature. The relative density of bands was analysed on an Odyssey infrared scanner (LI-COR Bioscience). The densitometry values were normalized with respect to the values of anti-histone 3 (H3, 1:1000, bioWORLD, Dublin, OH, USA) for Nrf2 or anti-β-actin (1:3000, Santa Cruz Biotechnology) for STAT5b, Keap1, HO-1 and NQO1 immunoreactivity.

### Immunohistochemistry and densitometric analysis

Liver tissue sections from histopathologic evaluation were also used for immunohistochemical analysis. Liver tissue sections from each of the three groups were placed on individual slides. After deparaffinization and hydration, sections were subjected to antigen retrieval (in 0.01 M citrate buffer, pH 6.0) by microwave for 30 min and immersed in 3% hydrogen peroxide in methanol for 30 min to abolish endogenous peroxidase activity. The sections were incubated with 5% normal goat serum to block non-specific binding. This procedure was followed by an overnight incubation with polyclonal rabbit anti-Nrf2 antibody (1:100, Abcam, ab31163), polyclonal rabbit anti-HO-1 antibody (1:100, Abcam, ab13243) and mouse anti-NQO1 monoclonal antibody (1:500, Abcam, ab28947) at 4 °C. After washing, the sections were incubated with biotinylated goat anti-mouse IgG (Jackson ImmunoResearch, 1:300) or goat anti-rabbit IgG (Jackson ImmunoResearch, 1:300) for 2 h at room temperature. Following the incubation of sections for 1 h at RT in horseradish peroxidase-conjugated streptavidin (1:300), the sections were stained for 5 min in a solution containing 0.05% diaminobenzidine (Sigma) and 0.03% H_2_O_2_ in 0.05 M Tris-HCl buffer (pH 7.6) to reveal immunoreactions^40^.

A computer-assisted image analysis system (Olympus, BX 61, Japan; Image-Pro Plus 6.0) was used for the average optical density (AOD) measurements of Nrf2, HO-1 and NQO1 immunoreactive intensity. Images were acquired after calibration of the system to eliminate saturation of grey levels for an accurate determination of optical density. The sections processed without primary antibody were used to determine the level of nonspecific staining for the entire experiment. After subtraction of the nonspecific staining, the AOD of Nrf2, HO-1 and NQO1 was measured. To prevent differences arising from variations in the conditions of tissue processing and densitometric analysis, all sections were simultaneously processed. All microscopic and computer parameters were kept constant throughout the study. Ten sections were selected from each rat. The averaged value of the AOD of Nrf2, HO-1 and NQO1 was presented for each rat^40^.

### Statistical analyses

We applied tests of normality (Kolmogorov–Smirnov test) and homogeneity of variance (Levene’s test) to all data. If both normal distribution (*P* > 0.1) and homogeneity of variance (*P* > 0.1) were found, then a parametric test was performed by one-way analysis of variance (one-way ANOVA) followed by a Student–Newman–Keuls (SNK) post hoc test for multiple comparisons. Otherwise, we used non-parametric statistics, namely the Kruskal–Wallis test, and if *P* < 0.05, post hoc analysis between groups were performed using the Mann–Whitney U test. Differences were considered to be significant when *P* values were less than 0.05. All of the data are presented as the mean ± SD^40^.

## Supplementary information

Supplementary information
