# Supplementary material for: Testosterone propionate activated the Nrf2-ARE pathway in ageing rats and ameliorated the age-related changes in liver
Source: Sci Rep. 2019 Dec 9;9:18619. doi: 10.1038/s41598-019-55148-0 (PMC6901587; doi:10.1038/s41598-019-55148-0)
Supplement: Supplementary file 1 — Supplementary information [file 41598_2019_55148_MOESM1_ESM.docx]

**Testosterone propionate activated the Nrf2-ARE pathway in ageing rats and ameliorated the age-related changes in liver**

Guoliang Zhang^1, 2^, Rui Cui^2^, Yunxiao Kang^1^, Chunxiao Qi^2^, Xiaoming Ji^1^, Tianyun Zhang^1^, Qiqing Guo^1^, Huixian Cui^2, 3^, Geming Shi^1, 3^

^1^ Department of Neurobiology, Hebei Medical University, Shijiazhuang, 050017, PR China; ^2^ Department of Human Anatomy, Hebei Medical University, Shijiazhuang, 050017, PR China; ^3^ Neuroscience Research Center, Hebei Medical University, Shijiazhuang, 050017, PR China; ^4^ Hebei Key Laboratory of Forensic Medicine, Department of Forensic Medicine, Hebei Medical University, Shijiazhuang, 050017, PR China.

Guoliang Zhang and Rui Cui contributed equally to this work.

Correspondence and requests for materials should be addressed to G. S. (email: shigeming@163.com).

**Supplementary Information**

S1**:** Orignial Western image for Figure 6a.







STAT5b β-actin







Keap1 β-actin







Nrf2 H3


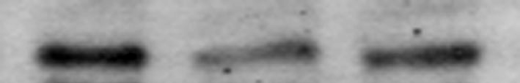




HO-1 β-actin







NQO1 β-actin
